# A role of p38 mitogen-activated protein kinase in adenosine A_1 _receptor-mediated synaptic depotentiation in area CA1 of the rat hippocampus

**DOI:** 10.1186/1756-6606-1-13

**Published:** 2008-10-23

**Authors:** Ying-Ching Liang, Chiung-Chun Huang, Kuei-Sen Hsu

**Affiliations:** 1 Department of Pharmacology, College of Medicine, National Cheng Kung University, No. 1, University Road, Tainan City 701, Taiwan; 2 Institute of Basic Medical Sciences, College of Medicine, National Cheng Kung University, No. 1, University Road, Tainan City 701, Taiwan

## Abstract

**Background:**

Although long-term potentiation (LTP) of synaptic strength is very persistent, current studies have provided evidence that various manipulations or pharmacological treatment when applied shortly after LTP induction can reverse it. This kind of reversal of synaptic strength is termed as depotentiation and may have a function to increase the flexibility and storage capacity of neuronal networks. Our previous studies have demonstrated that an increase in extracellular levels of adenosine and subsequent activation of adenosine A_1 _receptors are important for the induction of depotentiation; however, the signaling downstream of adenosine A_1 _receptors to mediate depotentiation induction remains elusive.

**Results:**

We confirm that depotentiation induced by low-frequency stimulation (LFS) (2 Hz, 10 min, 1200 pulses) was dependent on adenosine A_1 _receptor activation, because it was mimicked by bath-applied adenosine A_1 _receptor agonist *N*^6^-cyclopentyladenosine (CPA) and was inhibited by the selective adenosine A_1 _receptor antagonist 8-cyclopentyl-1,3-dipropylxanthine (DPCPX). Pretreatment of the hippocampal slices with the selective p38 mitogen-activated protein kinase (MAPK) inhibitors, 4-(4-fluorophenyl)-2-(4-methylsulfinylphenyl]-5-(4-pyrudyl)-1*H*-imidazole (SB203580) or *trans*-1-(4-hydroxycyclohexyl)-4-(fluorophenyl)-5-(2-methoxypyrimidin-4-yl)imidazole (SB239063), prevented the induction of depotentiation by LFS and CPA. In agreement with electrophysiological observation, both LFS- and CPA-induced depotentiation are associated with an increase in p38 MAPK activation, which are blocked by DPCPX or SB203580 application.

**Conclusion:**

These results suggest that activation of adenosine A_1 _receptor and in turn triggering p38 MAPK signaling may contribute to the LFS-induced depotentiation at hippocampal CA1 synapses.

## Background

Long-term potentiation (LTP) is a long-lasting form of activity-dependent synaptic plasticity that is generally thought to play crucial roles in learning and memory processes in the brain [1,2]. Although LTP is remarkable for its stability, numerous studies have revealed that it is initially labile and sensitive to disruption by a variety of interfering events and agents [3,4]. Such reversal of synaptic strength from the potentiated state to pre-LTP levels has been termed depotentiation and may provide a mechanism of preventing the saturation of the synaptic potentiation and increase the efficacy and the capacity of the information storage of the neuronal networks [3].

A sustained train of low-frequency stimulation (LFS) is a powerful paradigm for studying the molecular mechanism underlying the induction of depotentiation. Previous work from our and other laboratories have demonstrated that trains of 1–5 Hz LFS can produce an enduring and complete reversal of synaptic potentiation when delivered within 10 min of LTP induction [1,3,5,6]. So far, a number of synaptic signaling molecules have been identified that participates in the induction of depotentiation including adenosine, calcineurin, nitric oxide, and small GTPase Rap2 [6-12]. Less well understood are the processes that control signaling from these synaptic ligands and receptors. For example, we have previously proposed that the reversal of LTP at the Schaffer collateral-CA1 synapses by LFS is associated with an increase of extracellular levels of adenosine acting on adenosine A_1 _receptor to interrupt the cAMP/protein kinase A (PKA)-dependent biochemical processes leading to LTP [6,11]. We further demonstrated that the efflux of cAMP is the potential source for the increased extracellular adenosine underlying LFS-induced depotentiation [6]. However, it is still unclear the signaling downstream of adenosine A_1 _receptors to mediate the induction of LFS-induced depotentiation. Recent data suggest that p38 mitogen-activated protein kinase (MAPK) activation may contribute to adenosine A_1 _receptor-mediated synaptic depression in the hippocampal CA1 region [13]. Moreover, p38 MAPK signaling has been shown to be an important mediator of AMPA receptor surface trafficking during synaptic plasticity, a process crucial for rapid altering synaptic strength [14,15]. It became, therefore, of interest to study possible roles of p38 MAPK in adenosine A_1 _receptor-mediated depotentiation.

Here, we tested whether p38 MAPK signaling is involved in the induction of LFS-induced depotentiation in the CA1 region of hippocampus. Our study provides evidence that p38 MAPK may serve as a signaling downstream of adenosine A_1 _receptor activation to induce depotentiation.

## Results

### p38 MAPK contributes to LFS-induced depotentiation

**Figure 1:**
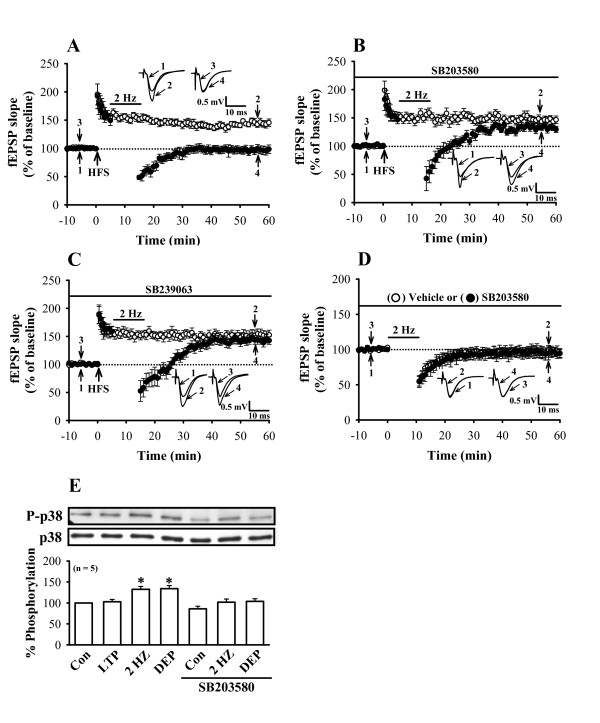
**p38 MAPK contributes the induction of LFS-induced depotentiation**. (A) Summary of experiments showing that LFS (2 Hz, 10 min) given 5 min after two trains of 100 Hz HFS almost completely reversed LTP (n = 8; ○), whereas fEPSPs in slices that received HFS without LFS exhibited persistent potentiation (n = 8; ●). (B) Summary of experiments showing that pretreatment of slices with the p38 MAPK inhibitor SB203580 (1 μM) blocked the induction of LFS-induced depotentiation (n = 8; ●) but had no effect on the induction of LTP (n = 6; ○). (C) Summary of experiments showing that pretreatment of slices with the p38 MAPK inhibitor SB239063 (1 μM) blocked the induction of LFS-induced depotentiation (n = 5; ●) but had no effect on the induction of LTP (n = 4; ○). (D) Summary of experiments showing that the protocol of LFS had no lasting effect on synaptic transmission in the presence of vehicle (n = 6; ○) or SB203580 (n = 6; ●). The superimposed fEPSP in the inset illustrates respective recordings from example experiments taken at the time indicated by *number*. *Horizontal bars *denote the period of delivery LFS or SB203580. (E) Representative immunoblots showing that the induction of LFS-induced depotentiation (DEP) is accompanied by a significant increase in p38 MAPK phosphorylation and that SB203580 (1 μM) prevented this enhancement action. Group data showing the normalization of phospho-p38 MAPK to the nonphosphorylated form was determined in each group of five experiments. In each experiment, 21–28 slices obtained from 2–3 rats were used. *, *p *< 0.05 (unpaired Student's t test) as compared with the control (Con) group.

Using field potential recordings, we first tested for the existence of a LFS-induced depotentiation in the CA1 region of hippocampal slices. LTP was induced by two 1-sec trains of 100 Hz stimuli separated by intertrain interval of 20 sec. The mean slope of the field excitatory postsynaptic potential (fEPSP) measured 50 min after high-frequency tetanic stimulation (HFS) was 146.5 ± 4.2% (n = 8) of baseline (Fig. 1A). To establish a reliable depotentiation, a long train of LFS protocol, 2 Hz/1200 pulse stimulation, was used [6,16]. As expected, when LFS was applied 5 min after LTP induction, LTP was almost completely reversed (Fig. 1A). The mean residual potentiation measured 40 min after the end of LFS was 96.5 ± 5.8% (n = 8) of baseline. Thus, these results generally confirmed previous studies showing that LTP is vulnerable to disruption by depotentiation stimuli within a short period after its induction [6,17,18].

To assess the role of p38 MAPK in the induction of LFS-induced depotentiation, the highly selective p38 MAPK inhibitor 4-(4-fluorophenyl)-2-(4-methylsulfinylphenyl]-5-(4-pyrudyl)-1*H*-imidazole (SB203580) was used. As shown in Figure 1B, SB203580 (1 μM) treatment alone had no effect on the induction of LTP. The mean slope of the fEPSP measured 50 min after HFS was 153.6 ± 8.5% (n = 6) of baseline, which was not statistically different from the corresponding values of control slices. In marked contrast to control slices, application of LFS, 5 min after LTP induction, failed to induce depotentiation in all eight slices tested. The mean residual potentiation measured 40 min after the end of LFS was 138.5 ± 6.2% (n = 8) of baseline (Fig. 1B). To further confirm a role of p38 MAPK in the induction of depotentiation, in another set of experiments, we examined the effect of another 38 MAPK inhibitor *trans*-1-(4-hydroxycyclohexyl)-4-(fluorophenyl)-5-(2-methoxypyrimidin-4-yl)imidazole (SB239063) on the LFS-induced depotentiation. We found that application of SB239063 (1 μM) failed to affect the LTP induction (148.5 ± 8.1% of baseline, n = 4; *p *> 0.05 when compared with control LTP slices; unpaired Student's *t *test), but prevented the LFS-induced depotentiation (Fig. 1C). The mean residual potentiation measured 40 min after the end of LFS was 139.5 ± 7.5% (n = 5) of baseline. In addition, in both control and SB203580-treated slices, 10 min of 2 Hz stimulation delivered to Schaffer collateral-CA1 synapses had no long-lasting effect on the synaptic transmission (Fig. 1D). The mean slope of the fEPSP measured 40 min after the end of LFS was 96.5 ± 6.7% (n = 6) and 98.3 ± 7.2% (n = 6) of baseline, respectively. Similar results were also obtained using an antibody that selectively recognizes phosphorylated, activated p38 MAPK [19]. As shown in Figure 1E, phospho-p38 MAPK was readily detected in untreated control slices, and the levels were increased to 132.5 ± 6.8% (n = 5) of control in LFS (2 Hz)-treated slices. However, no significant changes in the levels of phospho-p38 MAPK were observed 30 min after LTP induction (102.8 ± 5.6% of control slices, n = 5; *p *> 0.05; unpaired Student's *t *test). In LFS-induced depotentiation experiments, we found that there was significant increased the levels of phospho-p38 MAPK (134.2 ± 7.2% of control slices, n = 5; *p *< 0.05; unpaired Student's *t *test). In addition, pretreatment of the slices with SB203580 (1 μM) completely blocked the increase in p38 MAPK phosphorylation induced by LFS (Fig. 1E). These results strongly support the view that the p38 MAPK is an obligatory component of the biochemical bases that serves the induction of LFS-induced depotentiation.

### Adenosine A_1 _receptor-mediated depotentiation was decreased by p38 MAPK inhibition

**Figure 2:**
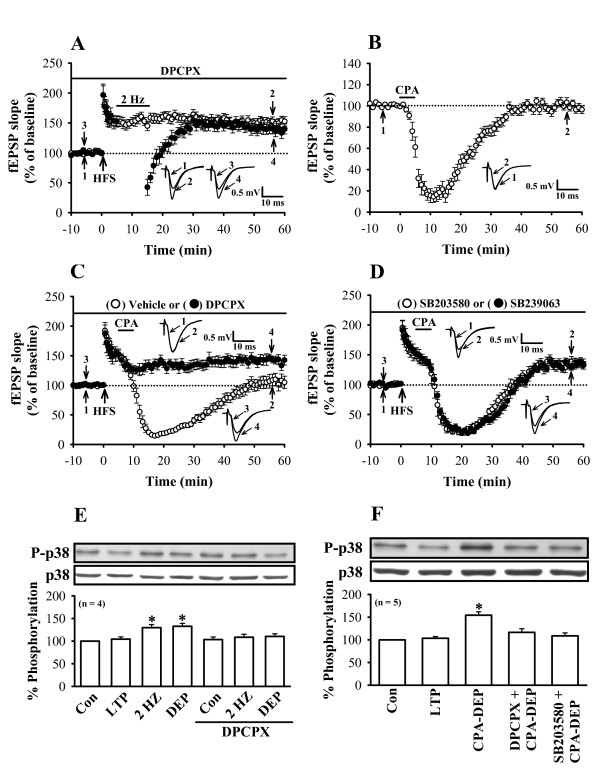
**Application of p38 MAPK inhibitor prevented the induction of adenosine A_1 _receptor-mediated depotentiation**. (A) Summary of experiments showing that pretreatment of slices with the adenosine A_1_receptor antagonist DPCPX (0.1 μM) blocked the induction of LFS-induced depotentiation (n = 5; ●) but had no effect on the induction of LTP (n = 6; ○). (B) Summary of experiments showing that application of CPA (0.2 μM) for 5 min produced a depression of fEPSPs (n = 6; ○). The fEPSP fully recovered after washout of drug. (C) Summary of experiments showing that CPA (0.2 μM; 5 min) given 5 min after two trains of 100 Hz HFS almost completely reversed LTP (n = 6; ○). Note that CPA-induced depotentiation was completely blocked by DPCPX (n = 6; ●). (D) Summary of experiments showing that pretreatment of slices with SB203580 (1 μM, n = 6; ○) or SB239063 (1 μM, n = 4; ●) blocked the CPA-induced depotentiation. (E) Representative immunoblots showing that the induction of LFS-induction depotentiation (DEP) is accompanied by a significant increase in p38 MAPK phosphorylation and that DPCPX prevented this enhancement action. Group data showing the normalization of phospho-p38 MAPK to the nonphosphorylated form was determined in each group of four experiments. In each experiment, 21–28 slices obtained from 2–3 rats were used. (F) Representative immunoblots showing that the induction of CPA-induced depotentiation (CPA-DEP) is accompanied by a significant increase in p38 MAPK phosphorylation. Note that CPA-induced increase in p38 MAPK phosphorylation was significantly blocked by DPCPX or SB203580. Group data showing the normalization of phospho-p38 MAPK to the nonphosphorylated form was determined in each group of five experiments. In each experiment, 15–20 slices obtained from 2 rats were used. *, *p *< 0.05 (unpaired Student's t test) as compared with the control (Con) group.

We and other laboratories have previously shown that an increase of extracellular adenosine acting on adenosine A_1 _receptors may be a component of the mechanism that triggers LFS-induced depotentiation in the hippocampal CA1 region [6,8,18]. A recent study has revealed that adenosine A_1 _receptor-mediated synaptic depression is dependent on p38 MAPK activation [13]. It was therefore of interest to examine the relationship between p38 MAPK activation and adenosine-mediated signaling pathways in the induction of depotentiation. We began by confirming that LFS-induced depotentiation can be blocked by the selective adenosine A_1 _receptor antagonist 8-cyclopentyl-1,3-dipropylxanthine (DPCPX). As shown in Figure 2A, the LFS-induced depotentiation was significantly inhibited in the presence of DPCPX (0.1 μM). The slope of fEPSP after LFS recovered close to the initial LTP levels. The mean residual potentiation measured 40 min after the end of LFS was 140.9 ± 8.6% (n = 5) of baseline. Application of DPCPX alone had no significant effect on the induction of LTP (149.7 ± 7.5% of baseline, n = 6; *p *> 0.05; unpaired Student's *t *test).

We next tested whether the reversal of LTP induced by a direct adenosine application was also affected by p38 MAPK antagonism. Because adenosine may act at multiple receptor subtypes and is rapidly degraded by ecto-nucleotidases [20], we used the specific adenosine A_1 _receptor agonist *N*^6^-cyclopentyladenosine (CPA) to address this issue. Application of CPA (0.2 μM) for 5 min potentially depressed the fEPSP by 84.5 ± 6.5% of baseline (n = 6). The fEPSP fully recovered after washout of drug (Fig. 2B). We next examined the effect of CPA application on the previously established LTP. In agreement with our previous findings [6,11], CPA (0.2 μM for 5 min) applied 5 min after LTP induction almost completely reversed LTP (Fig. 2C). The mean residual potentiation measured 40 min after washout of CPA was 98.9 ± 7.2% (n = 6) of baseline. When CPA was applied in the presence of DPCPX (0.1 μM), both acute response and the resulting depotentiation were inhibited significantly (Fig. 2C). The mean residual potentiation measured 40 min after washout of CPA was 142.3 ± 6.5% (n = 6) of baseline. In addition, we tested the effect of SB203580 and SB239063 on the induction of depotentiation by CPA. As shown in Figure 2D, treatment of either SB203580 (1 μM) or SB239063 (1 μM) had little effect on the acute response during CPA application but significantly blocked the induction of CPA-induced depotentiation. The mean residual potentiation measured 40 min after washout of CPA was 136.5 ± 6.7% (n = 6) and 132.3 ± 7.4% (n = 4) of baseline, respectively. In addition, pretreatment of the slices with DPCPX (0.1 μM) completely blocked the increase in p38 MAPK phosphorylation induced by LFS (Fig. 2E). Figure 2F shows that substantial increase in the levels of phospho-p38 MAPK is evident in CPA-induced depotentiation slices. Pretreatment of either DPCPX (0.1 μM) or SB203580 (1 μM) for 20 min also blocked the increase in p38 MAPK phosphorylation induced by CPA. Together, these results suggest that p38 MAPK seems to be the downstream signaling molecule for adenosine A_1 _receptor activation to induce depotentiation.

## Discussion

In this study, we identify a novel interaction between adenosine A_1 _receptor and p38 MAPK in hippocampal neurons. We have shown that activation of adenosine A_1 _receptors, either by a train of LFS or a brief application of adenosine A_1 _receptor agonist CPA, can trigger a depotentiation by a mechanism that requires the activation of p38 MAPK signaling pathway.

A number of studies have revealed an important role for extracellular adenosine acting on adenosine A_1 _receptors in mediating the induction of depotentiation [6,8,11,18]. However, the signaling downstream of adenosine A_1 _receptor activation to trigger depotentiation remains elusive. We have previously shown that activation of adenosine A_1 _receptor may interrupt the cAMP-PKA-dependent biochemical processes associated with the LTP expression [6,11]. Here, we have extended these findings by demonstrating that p38 MAPK may lie downstream of adenosine A_1 _receptor activation to induce depotentiation. Evidence supporting this prediction is that pretreatment of the hippocampal slices with the specific p38 MAPK inhibitors, SB203580 and SB239063, effectively prevented the induction of depotentiation by LFS and CPA. We further show that the induction of LFS- or CPA-induced depotentiation also leaded to a significant increase in the levels of p38 MAPK phosphorylation and these effects were prevented by prior treatment of the hippocampal slices with adenosine A_1 _receptor antagonist. Such a result is in line with the recent observation that p38 MAPK activation contributes to adenosine A_1 _receptor-mediated synaptic depression at Schaffer collateral-CA1 synapses [13].

The induction and expression of depotentiation involve multiple cellular and molecular mechanisms [3,4]. A number of studies indicate that the induction of depotentiation relies on synaptic activation of NMDA receptors [5,11,21] or metabotropic glutamate (mGlu) receptors [22-24]. We have previously shown that hippocampal CA1 depotentiation induced by LFS (2 Hz, 10 min, 1200 pulses) is dependent on NMDA receptor activation [11]. We further showed that activation of NMDA receptors may stimulate adenosine-mediated signaling pathways to trigger depotentiation [11]. In this study, we have extended theses findings by showing that p38 MAPK could act downstream of adenosine A_1 _receptor activation to undergo the induction of depotentiation. The role of mGlu receptors in the induction of depotentiation remains controversial, largely because some groups have been unable to replicate the findings that mGlu receptors are necessary for depotentiation induction under their experimental conditions [25]. The reason for this discrepancy is not clear but could be attributable partly to the use of different depotentiating stimulation protocols. Because the ability of LFS used in the present study to erase LTP was not significantly affected by either non-selective mGlu antagonist α-methyl-4-carboxyphenylglycine (MCPG) or selective group 1 mGlu receptor antagonist 2*S*-2-amino-2-(1*S*,2*S*-2-carboxycycloprop-1-yl)-3-(xanth-9-yl)-propanoic acid (LY341495) treatment (Huang and Hsu unpublished observation), we suggest that the activation of mGlu receptors is not required for the LFS-induced depotentiation in the hippocampal CA1 region.

How might adenosine A_1 _receptors stimulation lead to p38 MAPK activation? The signal transduction events that couple adenosine A_1 _receptor stimulation to p38 MAPK activation were not examined here. A previous study in the smooth muscle cell line has indicated that p38 MAPK can be activated by the adenosine A_1 _receptors via a pertussis toxin-sensitive signaling pathway [26]. It seems likely that the p38 MAPK activation by the adenosine A_1 _receptors is mediated by G_i/o _proteins coupling to classical intracellular signaling pathways such as modulation of cAMP production or the phospholipase C pathway [27,28]. Interestingly, several other studies have reported that G protein-coupled receptors can also activate p38 MAPK via Gβγ subunits. For example, in human embryonic kidney 293 cells, G_i_-coupled m2 muscarinic receptors and G_s_-coupled β-adrenergic receptors were found to stimulate p38 MAPK via Gβγ [29]. Similarly, our previous study has also demonstrated that by releasing Gβγ subunits, the G_q/11_-coupled type 5 metabotropic glutamate receptors can activate small GTPase Rap1 to mediate (S)-3,5-dihydroxyphenylglycine (DHPG)-induced p38 MAPK activation in hippocampal CA1 neurons [15]. According to these previous observations, it will be important for future studies to identify which of these mechanisms is responsible for the adenosine A_1 _receptor-mediated p38 MAPK activation observed in hippocampal CA1 neurons.

How might p38 MAPK activation reverse the previously established LTP? A most likely possibility is that a decrease in the surface expression of AMPA receptors contributes to this process. Activity-dependent changes in AMPA receptor trafficking have been proposed to play a critical role in mediating bidirectional synaptic plasticity [30,31]. It has been shown that LTP induction in hippocampal slices is associated with an increase in the delivery of AMPA receptors to dendritic spines [32,33], whereas the expression of LTD is associated with a decrease in the surface expression of AMPA receptors [34,35]. With the use of biochemical surface biotinylation assay, we have previously shown that the expression of DHPG-induced depotentiation is associated with a reduction in the increase of the surface expression of AMPA receptors seen with LTP [24]. This finding strongly implies that the removal of synaptic surface AMPA receptor is a potential mechanism underlying the expression of depotentiation in hippocampal neurons. In addition, previous work from our and other laboratories has demonstrated that activation of p38 MAPK may lead to synaptic removal of surface AMPA receptors [12,15]. These results suggest that p38 MAPK-mediated synaptic removal of surface AMPA receptors may account for, at least in part, the development of adenosine A_1 _receptor-dependent form of depotentiation. Our previous studies have found that the expression of LFS-induced depotentiation was accompanied by a persistent dephosphorylation of the GluR1 subunit of AMPA receptors at serine 831, a protein kinase C and calcium/calmodulin-dependent protein kinase II substrate, but not at serine 845, a substrate of PKA [6,11]. Activation of protein phosphatase 1 (PP1) triggered this dephosphorylation (Huang et al., 2001) and inhibition of PP1 activity rendered the depotentiation stimuli ineffective [6,36]. Taken together, these findings indicate that the interaction between p38 MAPK and PP1 can determine whether depotentiation is induced at Schaffer collateral-CA1 synapses.

Our results indicate that SB203580 produced only little effect on the acute response during CPA application but significantly blocked the induction of CPA-induced depotentiation. This finding is in contrast with observation made in a previous study [13], which reported that SB203580 potently reduced CPA-mediated synaptic depression. The reason for this discrepancy is not clear. It remains to be determined if the use of different concentrations of CPA (0.2 μM vs. 40 nM) and SB203580 (1 μM vs. 25 μM) has a role in explaining the conflicting results. In addition, SB203580 has been shown to effectively compete the binding of adenosine A_1 _receptor antagonist DPCPX in rat brain membrane at micromolar concentrations [37]. Given that the concentration of SB203580 used in this study was below the effective concentration as adenosine A_1 _receptor antagonist [37], we therefore assumed that the effect of SB203580 on depotentiation was mediated via the inhibition of p38 MAPK.

An intriguing observation of this study is that 2 Hz stimulation alone in control slices significantly increased the levels of phospho-p38 MAPK but had no long-lasting effect on the basal synaptic transmission (Fig. 1D, 1E and 2E). A plausible explanation for this finding may be that p38 MAPK activity is required but is not sufficient for the induction of synaptic depotentiation or depression. However, further experiments are required to test this possibility.

## Conclusion

In summary, we have shown in this study that p38 MPAK is activated by adenosine A_1 _receptor stimulation and contributes to the induction of depotentiation in the hippocampal CA1 region. Given the potential role of LTP in learning and memory formation and the processes disrupting the formation of stable LTP may be the possible cause for the loss of memory [1,3], our findings imply a possibility that p38 MAPK is a potential common signaling molecule to exert the mechanism of memory loss. It would be interesting to explore whether the selective p38 MAPK inhibitor may be implicated in enhancing cognition in the treatment of some dementia disorders.

## Methods

### Hippocampal slice preparation

All procedures were performed according to NIH guidelines for animal research (Guide for the Care & Use of Laboratory Animals, NRC, 1996) and approved by the Institutional Animal Care and Use Committee at National Cheng Kung University. Hippocampal slices were obtained from 28- to 35-day old young male Sprague-Dawley rats for extracellular recordings by the procedures described previously [6]. In brief, animals were anesthetized with halothane and decapitated with guillotine, and the hippocampi were removed, placed in ice cold artificial CSF (aCSF) solution and cut with a Leica VT1000S tissue slicer (Leica, Nussloch, Germany) into transverse slices of 400 μm. After their preparation, slices were placed in a holding chamber of aCSF oxygenated with 95% O_2_-5% CO_2 _and kept at room temperature for at least 1 h before used. The composition of the aCSF solution was (in mM): NaCl 117, KCl 4.7, CaCl_2 _2.5, MgCl_2 _1.2, NaHCO_3 _25, NaH_2_PO_4 _1.2 and glucose 11 at pH 7.3–7.4 and equilibrated with 95% O_2_-5% CO_2_.

### Electrophysiological recordings

For the extracellular field potential recordings, a single slice was transferred to a submerge-type recording chamber and held between two nylon nets. The chamber consisted of a circular well of a low volume (1–2 ml) and was continuously perfused with oxygenated aCSF at a flow rate of 2–3 ml/min at 32.0 ± 0.5°C. Area CA3 was surgically removed after sectioning. Extracellular field potential recordings were carried out using an Axoclamp-2B amplifier (Axon Instruments, Union City, CA). Microelectrodes were pulled from microfibre 1.0 mm capillary tubing on a Brown-Flaming electrode puller (Sutter Instruments, San Rafael, CA). The responses were low pass filtered at 2 kHz, digitally sampled at 5–10 kHz, and analyzed using pCLAMP software (Version 7.0; Axon Instruments). The evoked postsynaptic responses were induced in CA1 stratum radiatum by stimulation (0.02 ms duration) of Schaffer collateral/commissural afferents at 0.033 Hz with a bipolar stainless steel stimulating electrode. The stimulation strength was set to elicit a response having amplitude that was 30–40% of the maximum spike-free response. fEPSPs were recorded with a glass pipette filled with 1 M NaCl (2–3 MΩ resistance) and fEPSP slope was measured from approximately 20–70% of the rising phase using a least-squares regression. The LTP was induced by HFS, at the test pulse intensity, consisting of two 1-sec trains of stimuli separated by an intertrain interval of 20 sec at 100 Hz. Depotentiation was induced by application of 10 min low-frequency trains of stimuli at 2 Hz, and the stimulation intensity was the same as the test pulse intensity. All values of residual potentiation reported here were calculated as the changes in the slope of fEPSPs measured 40 min after the end of LFS.

### Western blotting

Hippocampal slices were prepared and treated with HFS with or without LFS exactly as described in the electrophysiological experiments. At the end of experiments, the CA1 subregion of the hippocampal slices between the positions of the stimulating and recording electrodes was dissected out and immediately frozen on dry ice. Three to four microdissected CA1 subregions (from control, 30 min after LTP induction, 15 min after the end of LFS, 20 min after washout of CPA) were pooled together. In each experiment, an entire set of control, LTP, or depotentiation pooled slices was taken from one animal. The microdissected subregions were lysed in ice-cold Tris-HCl buffer solution (TBS; pH 7.4) containing a cocktail of protein phosphatase and proteinase inhibitors (50 mM Tris-HCl, 100 mM NaCl, 15 mM sodium pyrophosphate, 50 mM sodium fluoride, 1 mM sodium orthovanadate, 5 mM EGTA, 5 mM EDTA, 1 mM phenylmethylsulfonyl fluoride, 1 μM microcystin-LR, 1 μM okadaic acid, 0.5% Triton X-100, 2 mM benzamidine, 60 μg/ml aprotinin, and 60 μg/ml leupeptin) to avoid dephosphorylation and degradation of proteins, and ground with a pellet pestle (Kontes glassware, Vineland, NJ, USA). Samples were sonicated and spun down at 15,000 × g at 4°C for 10 min. The supernatant was then assayed for total protein concentration using Bio-Rad Bradford Protein Assay Kit (Hercules, CA). Each sample was separated in 10% SDS-PAGE gel. Following the transfer on PVDF membranes, blots were blocked in buffer solution containing 5% milk and 0.1% Tween-20 in PBS (124 mM NaCl, 4 mM KCl, 10 mM Na_2_HPO_4_, and 10 mM KH_2_PO_4_, pH 7.2) for 1 h and then blotted for 2 h at room temperature with antibodies that recognize phosphorylated p38 MAPK (1:1000; New England BioLabs, Beverly, MA). It was then probed with HRP-conjugated secondary antibody for 1 h and developed using the ECL immunoblotting detection system (Amersham Biosciences, Buckinghamshire, UK), according to manufacturer's instructions. The immunoblots using phosphorylation site-specific antibodies were subsequently stripped and reprobed with an antibody that recognizes p38 MAPK (1:500; New England BioLabs). Immunoblots were analyzed by densitometry using Bio-profil BioLight PC software. Only film exposures that were in the linear range of the ECL reaction were used for quantification analysis.

### Drug application

All drugs were applied by manually switching the superfusate. Drugs were diluted from stock solutions just before application. CPA was prepared by first dissolving it in an equimolar amount of HCl as a concentrated stock and then diluting to its final concentration in aCSF. Other drugs used in this study were dissolved in distilled water. SB203580, SB239063 and CPA were purchased from Tocris Cookson (Bristol, UK).

### Statistical analysis

All data are expressed as means ± SEM and the statistic significance was determined using the Mann-Whitney *U*-test or Student's *t*-test. Numbers of experiments are indicated by n. Probability values of *p *< 0.05 were considered to represent significant differences.

## Competing interests

The authors declare that they have no competing interests.

## Authors' contributions

YCL and CCH performed the experiments and the statistical analysis, YCL, CCH, and KSH designed the study and wrote the manuscript. All authors read and approved the final manuscript.
